# Novel Trinorditerpene from *Dysoxylum parasiticum* (Osbeck) Kosterm: Leaf Extract with Cytotoxic, Antioxidant and α-Glucosidase Inhibitory Activities

**DOI:** 10.3390/molecules30244747

**Published:** 2025-12-12

**Authors:** I Putu Agus Hendra Wibawa, Faris Hermawan, Minarti, Puspa Dewi Lotulung, Nina Artanti, Muhammad Hanafi, Vito M. Butardo, Peter J. Mahon

**Affiliations:** 1 School of Science, Computing and Emerging Technologies, Swinburne University of Technology, Hawthorn, VIC 3122, Australia; iput002@brin.go.id (I.P.A.H.W.); vbutardo@swin.edu.au (V.M.B.J.); 2 Research Center for Applied Botany, National Research and Innovation Agency (BRIN), Kawasan Sains dan Teknologi Dr. (H.C.) Ir. Soekarno, Cibinong, Bogor 16122, Indonesia; 3 Research Center for Pharmaceutical Ingredients and Traditional Medicine, National Research and Innovation Agency (BRIN) KST BJ Habibie Puspiptek Area, Tangerang Selatan 15314, Indonesia; faris025@brin.go.id (F.H.); mina002@brin.go.id (M.); puspade@yahoo.com (P.D.L.); muha002@brin.go.id (M.H.); 4 Research Center for Molecular Chemistry, National Research and Innovation Agency (BRIN) KST BJ Habibie Puspiptek Area, Tangerang Selatan 15314, Indonesia; ninaartanti@gmail.com; 5 Magister Program at Faculty of Pharmacy, Pancasila University, Jakarta Selatan 12640, Indonesia

**Keywords:** cytotoxic, antioxidant, anti-diabetic, α-glucosidase

## Abstract

Natural products derived from plants have been extensively developed as alternative medicines due to their relatively minimal side effects. Here we present the purification and characteristics of parasitic acid, a novel trinorditerpene from *Dysoxylum parasiticum* leaf extract, and investigate various bioactivities. The structure of this compound was elucidated using extensive spectroscopic techniques, including 1D and 2D NMR, and high-resolution mass spectrometry, which revealed a unique trinorditerpene skeleton featuring a 3-carboxyfuran moiety. The purified trinorditerpene exhibited cytotoxicity against MCF-7 (IC_50_ 29.0 ± 0.8 μg/mL), antioxidant effects in the DPPH radical scavenging assay (IC_50_ 10.91 ± 0.04 μg/mL), and inhibition of α-glucosidase enzyme (IC_50_ 36 ± 1 μg/mL). Docking studies were also undertaken to explore the binding activities. This is the first report of a trinorditerpene-type diterpene from *D. parasiticum* with this unique combination of biological activities.

## 1. Introduction

In 2021, diabetes mellitus (DM) was responsible for 6.7 million fatalities annually, ranking as the sixth highest cause of death worldwide. Thirty-seven million people in the US suffer from DM. The American Diabetes Association lists Type 2 DM (T2DM) as the most rapidly increasing disease worldwide, and the fatality rate is anticipated to increase nearly tenfold by 2030 [1]. Cancer is the second leading cause of fatality in the United States, accounting for nearly 600,000 fatalities in 2021 [2,3]. Cancer is also ranked as one of the leading causes of death in the world, accounting for one in every six fatalities [1]. This is expected to expand year after year [4]. T2DM has been linked to an increased risk of numerous forms of cancer [5]. Treatment of this disease is very costly, estimated at around $379 billion per year in the US [1].

Conventional (medical) treatment drugs have been developed that can be used to treat T2DM and cancer. However, long-term use of chemical drugs can cause various side effects [6,7]. As such, natural plant medicines can be used as an alternative therapy option due to their minimal side effects. Several studies have also shown that antioxidant supplements have beneficial effects on T2DM and certain cancers [8,9,10].

Natural products (NPs) have a long history as a source of drug discovery; more than half of approved medications are derived from NPs, which also offer numerous benefits in the clinical treatment of complex disorders [11]. Compared to single-target medicines, NPs often have broader pharmacological activity and can overcome drug resistance produced by single-target inhibition [12]. The efficacy of several NP extracts has been established; nevertheless, given the complexity of their composition, identifying active substances and mechanisms of action remains a significant challenge.

Meliaceae is a flowering plant family of mostly trees and shrubs (and a few herbaceous plants, mangroves) in the order Sapindales. This tribe includes 50 genera and more than 1400 species, with a pantropical distribution. Most of its members are useful for their wood, fruit, or chemical content. Some of the pharmacological activities of this family are extremely important, including anticancer, antioxidant, antibacterial, anti-inflammatory, and hepatoprotective properties. This family also has antihelmintic, laxative, CNS depressant, thrombolytic, analgesic, and antimalarial properties. In addition, this family has insecticidal, antifeedant, repellent, and allelopathic effects [13].

*Dysoxylum parasiticum* (Osbeck) Kosterm is a large evergreen tree that belongs to the Meliaceae family, native to Southeast Asia and the Western Pacific region. This tree has characteristic aromatic wood and is known by Balinese ethnic groups in Indonesia as “majegau” or divine tree. The tree has been used traditionally for various purposes, such as construction, furniture-making, and in traditional medicine [14].

The leaf extract of *D. parasiticum* showed potential antiproliferative activity in previous studies [15]. Sesquiterpene compounds extracted from its bark have been proven to be cytotoxic against MCF-7 breast cancer cells [14]. Furthermore, various secondary substances with cytotoxic properties were collected from their leaves. Some of the compound compositions indicate that they are distinct sesquiterpene phenol dimers [16]. Several new compounds have been discovered from *D. parasiticum* leaf extract, such as three new dimeric sesquiterpene phenols, which showed cytotoxicity against human promyelocytic leukemia cells [16], and two new trimeric sesquiterpene phenols, which have cytotoxic activity against HL60 cells [17]. Moreover, two unidentified sesquiterpenoids, dysoticans A and B, have been discovered from the stem bark of *D. parasiticum* collected from West Java, Indonesia. These compounds showed moderate activity against human breast cancer MCF-7 and cervical cancer HeLa cells [18].

The entire chemical composition of *D. parasiticum* has yet to be fully characterized and it is possible to discover novel bioactive metabolites in this understudied species. The general objectives of this study were to contribute to the knowledge of the chemical constituents of *D. parasiticum*, identify new bioactive compounds with potential therapeutic applications, and provide a basis for further investigation into their mechanisms of action and structure-activity relationships.

Trinorditerpenes are diterpene derivatives that occur naturally in several plant species, such as the roots of *Flueggea virosa* and *Celastrus angulatus* [19,20]. Several reports have indicated that certain trinor-diterpenes exhibit potent cytotoxicity against human tumor cell lines, including A549, MDA-MB-231, HCT116, and BEL7404 [20]. Other trinorditerpenes have also been reported to display inhibitory activity against HCVcc infection and to exhibit cytotoxicity toward the Huh7.5 cell line [19]. Herein, we report the isolation of a novel trinor-diterpene, which we have named parasitic acid (12-(3-carboxyfuran-2-yl)-trinorlabda-5, 9-dien-15-oic acid), along with its cytotoxicity against MCF-7, antioxidant and α-glucosidase inhibitory activities.

## 2. Results and Discussion

**Figure 1:**
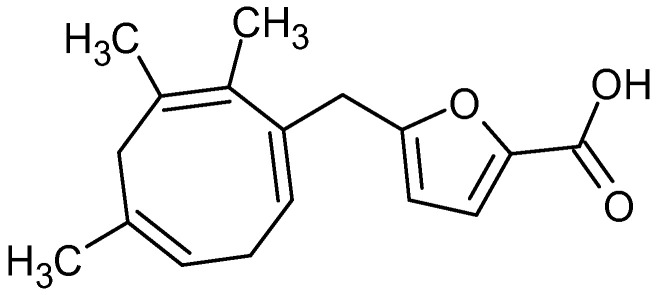
Structure of parasitic acid.

A novel trinorditerpene (12-(3-carboxyfuran-2-yl)-trinorlabda-5,9-dien-15-oic acid), designated in this study as parasitic acid, was isolated from the ethyl acetate extract of *D. parasiticum* leaves. Figure 1 shows the structure of parasitic acid as elucidated using extensive spectroscopic techniques, including 1D and 2D NMR, as well as high-resolution mass spectrometry as described in the Supporting Information. Trinorditerpene are diterpene derivatives whose carbon skeletons have been modified through the loss of three carbon atoms, typically from a side chain. A standard diterpene contains twenty carbon atoms (C_20_), formed from four isoprene (C_5_) units. Consequently, trinorditerpene generally possesses seventeen carbon atoms (C_17_), reflecting the removal of three carbon atoms from the original diterpene framework [19,20].

### 2.1. Cytotoxic Activity

**Figure 2:**
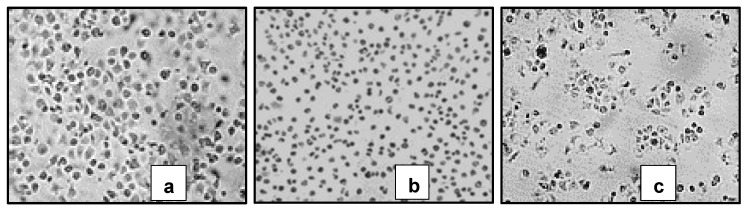
Themorphology of MCF-7 cells treated with (**a**) control cells, (**b**) Erlotinib treatment and (**c**) Parasitic acid. (**a**) Normal cells. (**b**,**c**) Cells showing characteristics of apoptosis (shrinkage & formation of apoptotic bodies). Viewed under an INV100 microscope (200×).

Parasitic acid showed significant cytotoxic activity against the breast cancer cell line MCF7 (Table 1). When compared with cells that grow normally, with the same magnification, the compound treatment shows that the cells have abnormal morphology and are damaged (Figure 2).

Microscopic examination revealed that treated cells exhibited marked morphological changes, including cell shrinkage, membrane blebbing, and reduced adherence compared to untreated controls (Figure 2), suggesting the induction of apoptotic cell death [21]. The cytotoxic potency of parasitic acid (IC_50_ 29.0 ± 0.8 μg/mL) is moderate compared to standard chemotherapeutic agents like doxorubicin, which typically has an IC_50_ of 0.2 to 10 μg/mL against MCF-7 [22,23,24]. However, its potential to act through multiple mechanisms, as evidenced by its concurrent antioxidant activity, suggests it might have advantages in terms of drug resistance or combination therapy applications. The biological activity of a compound is greatly influenced by its molecular structure. Previous studies have reported that the structure–activity relationship of diterpenes indicates that the presence of electron-withdrawing groups at positions C-15 and C-3 tends to enhance cytotoxic activity [25,26].

A significant limitation of the current study is the lack of cytotoxicity data against normal cells, which is crucial for determining the selectivity of the compound towards cancer cells. Future studies should evaluate parasitic acid against normal breast epithelial cells (e.g., MCF-10A) to establish a therapeutic index. Additionally, mechanistic studies including apoptosis assays (e.g., Annexin V/PI staining), cell cycle analysis, and measurement of ROS levels would help elucidate the precise mode of cell death.

**Figure 3:**
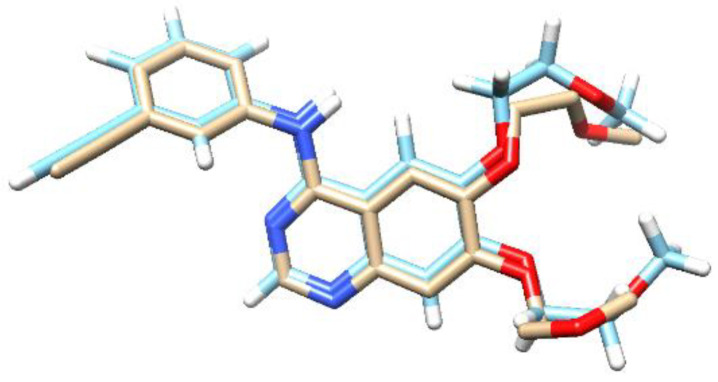
The overlapping structure of erlotinib of the X-ray Crystal Structure (light blue) with the docking result (white). The red regions correspond to oxygen atoms and the dark blue are nitrogens.

The molecular docking study of the compound parasitic acid was carried out on the Epidermal Growth Factor Receptor (EGFR) protein. EGFR protein is involved in cell growth and progression, including the promotion of angiogenesis, proliferation, metastasis, and inhibition of the apoptosis process. It was reported that high levels of EGFR have also been found in a variety of tumors, such as breast, prostate, ovarian, colorectal, and gastric [27]. EGFR Inhibitors, such as erlotinib, are able to block the activation of EGFR proteins [28]. These inhibitors interact with the ATP-binding site through a hydrogen bond, thereby blocking signal transduction [29]. The parasitic acid was docked into the protein’s active site to investigate potential interactions as protein inhibitors. To reach that goal, redocking was initially performed to validate the binding position and also to adjust the parameters of the docking process [30]. Erlotinib, as the native ligand of EGF, showed the RMSD value of 0.93 Å with the binding energy of −8.27 kcal/mol. The superimposed structure of erlotinib prior to docking and the results obtained from the redocking calculation are presented in Figure 3. The successful redocking of the native ligands, with RMSD values below 2.0 Å, validated the experimental parameters for the subsequent docking of parasitic acid [31].

**Figure 4:**
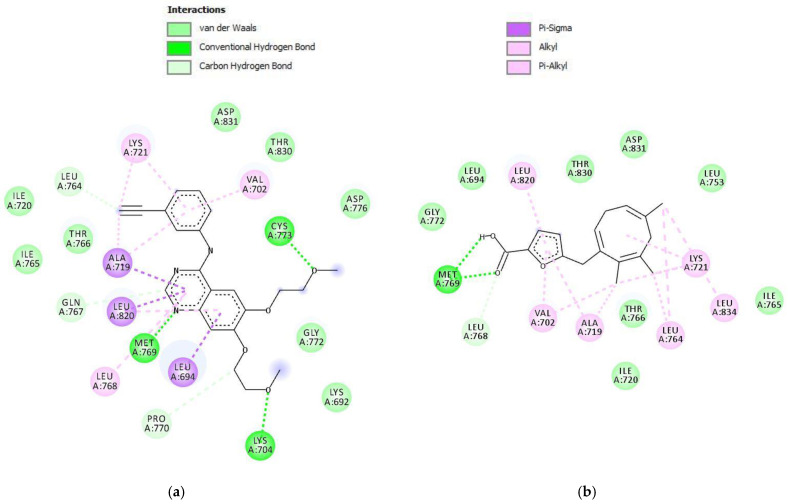
Intermolecular interactions (2D) of (**a**) Erlotinib and (**b**) parasitic acid in the active site of the EGFR protein.

The molecular docking result against the EGFR protein (Table 2) showed that parasitic acid has a binding energy of −8.18 kcal/mol. Compared with the native ligand erlotinib, the parasitic acid binding energy is higher. This result suggested that the inhibition activity toward the EGFR protein was weaker than erlotinib. These results are consistent with the in vitro assay, which demonstrated that erlotinib exhibited a lower IC_50_ compared to parasitic acid. Parasitic acid exhibited interactions with the EGFR protein through hydrogen bonding, van der Waals forces, carbon–hydrogen interactions, π–sigma, and π–alkyl interactions (Figure 4). Erlotinib established multiple stable interactions with key active site residues, including Lys721, Ala719, Leu820, Met769, Leu694, and Cys773, through a combination of hydrogen bonding and hydrophobic contacts. In contrast, parasitic acid exhibited fewer interactions, primarily involving Lys721, Ala719, Thr766, Leu820, Met769, and Val702, and notably lacked the hydrogen bond with Cys773 observed in the erlotinib complex. Collectively, these findings indicate that erlotinib demonstrates a more extensive and stable binding affinity toward the target protein compared to parasitic acid.

### 2.2. Antioxidant Activity

The antioxidant activity of parasitic acid was evaluated using the DPPH free radical scavenging assay. This compound exhibited considerable radical scavenging potential. At concentrations of 5, 10, and 25 µg/mL, parasitic acid demonstrated radical scavenging activities of 26.58%, 53.51%, and 92.93%, respectively, indicating a clear dose–response relationship. The difference in activity between quercetin, with an IC_50_ value of 6.1 ± 0.1 µg/mL, and parasitic acid, with an IC_50_ value of 10.91 ± 0.04 µg/mL, was statistically significant (*p* < 0.001) (Table 3). Parasitic acid exhibited antioxidant properties, and antioxidants have been reported to play an important role in protecting the body against various pathologies associated with oxidative stress, including diabetes mellitus and its related complications [32].

The strong antioxidant capacity within the diterpene framework can be attributed to several structural features. The presence of conjugated double bonds that facilitate electron delocalization, an electron-rich furan ring system, and a carboxylic acid group capable of donating hydrogen atoms all play crucial roles in enhancing its activity [33,34]. Parasitic acid is proposed to act through two complementary antioxidant mechanisms: hydrogen atom transfer (HAT) and single-electron transfer (SET). In the HAT mechanism, a hydrogen atom donated by the –OH group neutralizes free radicals, while in the SET mechanism, an electron donated by the compound converts reactive radicals into their inactive forms. Together, these mechanisms contribute to the compound’s overall antioxidant potential [35]. The antioxidant activity of this compound may be highly relevant to its potential therapeutic applications against oxidative stress associated with various diseases, such as cancer and diabetes. This suggests that parasitic acid may provide broad-spectrum protection against diverse types of free radicals and reactive oxygen species [36,37,38,39,40,41,42,43,44,45,46,47,48]. Further mechanistic studies employing electron spin resonance spectroscopy would be valuable to confirm these proposed mechanisms; however, the precise molecular mechanism remains to be elucidated.

### 2.3. α-Glucosidase Enzyme Inhibition

Parasitic acid exhibited α-glucosidase inhibitory activity with an IC_50_ value of 36.29 ± 1.06 µg/mL, indicating strong activity, although it was less potent compared to the reference compound quercetin (IC_50_ = 1.29 ± 0.06 µg/mL). The compound demonstrated a concentration-dependent inhibition pattern, with inhibition percentages of 0.39%, 2.32%, 15.12%, and 28.18% at concentrations of 5, 10, 20, and 25 µg/mL, respectively (Table 4). The analysis revealed a t-value of t (2.01) = −60.01, *p* < 0.001, indicating a highly significant difference between quercetin and parasitic acid. This strong α-glucosidase inhibitory activity of parasitic acid suggests its potential as an anti-diabetic agent, as α-glucosidase inhibitors are commonly used in the management of type 2 diabetes [39].

An ideal anti-diabetic agent should combine the properties of an α-glucosidase inhibitor with those of an antioxidant [40]. The α-glucosidase inhibitory activity of parasitic acid can be attributed to its unique structural features. The presence of a diterpene skeleton with both hydrophobic and hydrophilic regions suggests the compound may interact with the enzyme’s active site through multiple binding modes [39]. The furan ring and carboxylic acid groups likely form hydrogen bonds with key amino acid residues in the enzyme’s catalytic pocket, while the hydrophobic diterpene core could engage in van der Waals interactions with nonpolar regions of the binding site as observed in other studies [41,42]. This type of dual interaction pattern has been observed in other natural product α-glucosidase inhibitors [39,43,44,45,46]. Additionally, the compound’s demonstrated antioxidant properties may contribute to its anti-diabetic potential through a complementary mechanism, as oxidative stress is implicated in the pathogenesis of diabetic complications [47]. While the IC_50_ value suggests moderate inhibitory potency, the compound’s dual antioxidant and α-glucosidase inhibitory activities make it an interesting lead for further development of anti-diabetic agents.

These findings represent a significant contribution to natural product drug discovery, particularly in the context of multi-target therapeutic agents. The simultaneous presence of cytotoxic, antioxidant, and anti-diabetic properties in a single molecule is especially noteworthy, as it suggests potential applications in treating complex diseases where multiple pathological processes are involved. Future research directions should focus on: (1) detailed mechanistic studies using molecular and computational approaches to understand structure-activity relationships, (2) synthesis of structural analogs to optimize biological activities, (3) evaluation of in vivo efficacy and safety in relevant disease models, and (4) investigation of potential synergistic effects when combined with existing therapeutic agents. These studies will be crucial in developing parasitic acid as a lead compound for novel pharmaceutical applications, particularly in the treatment of cancer and metabolic disorders.

**Figure 5:**
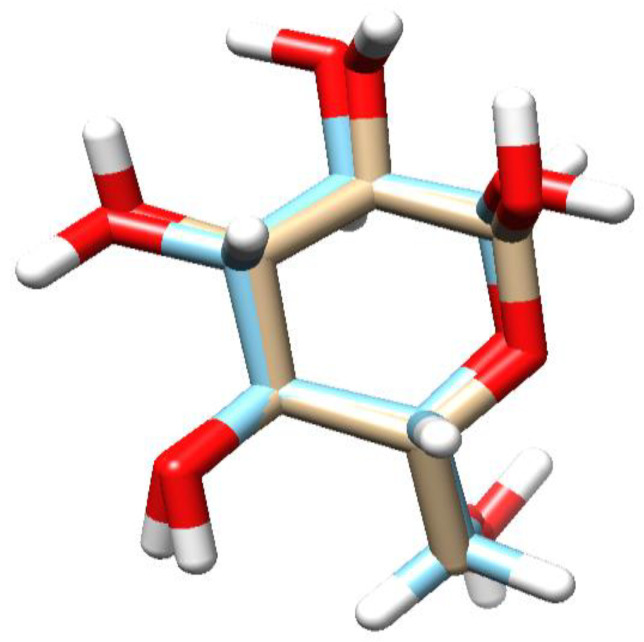
The overlapping structure of α-glucopyranose of the X-ray Crystal Structure (light blue) with the docking result (white). The red regions correspond to oxygen atoms.

Molecular docking study for parasitic acid was performed on the protein *S. cerevisiae* isomaltase. This protein is a common protein used for α-glucosidase docking [48]. The inhibition of α-glucosidase is a well-established therapeutic strategy in diabetes management, aimed at reducing hepatic glucose production and enhancing insulin sensitivity or secretion [49]. Among these approaches, α-glucosidase inhibition manages post-prandial hyperglycemia by preventing the enzymes from cleaving 1,4-glucosidic linkages in starch, thereby delaying its hydrolysis [50]. The parasitic acid was docked into the active site of each protein to study the possible interaction as a protein inhibitor agent. To reach that goal, we first performed the redocking process to validate the binding position and also to adjust the parameters of the docking process [30]. α-glucopyranose, as the native ligand of *S. cerevisiae* isomaltase protein, exhibited the RMSD value of 0.81 Å with the binding energy of −6.69 kcal/mol. The superimposed structure of α-glucopyranose prior to docking and the results obtained from the redocking calculation are presented in Figure 5. Redocking of these native ligands was successfully performed as the RMSD values were less than 2 Å, demonstrating that the experimental parameters were accurate enough to be used for the docking process of parasitic acid [31].

**Figure 6:**
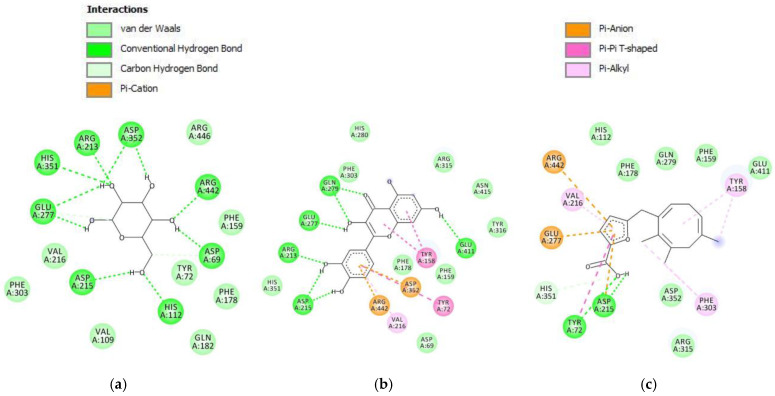
Intermolecular interactions (2D) of (**a**) α-glucopyranose, (**b**) quercetin, (**c**) parasitic acid in the active site of *S. cerevisiae* isomaltase protein.

The molecular docking result towards *S. cerevisiae* isomaltase protein showed that the compound parasitic acid has the binding energy value of −5.28 kcal/mol (Table 5). The binding energy of parasitic acid was higher than that of α-glucopyranose and quercetin. This result showed that the inhibitory effect against the *S. cerevisiae* isomaltase protein was weaker than that of α-glucopyranose and quercetin. These results align with the in vitro assay, demonstrating that quercetin has a lower IC_50_ relative to parasitic acid. Parasitic acid exhibited interactions with the *S. cerevisiae* isomaltase protein through hydrogen bonding, van der Waals forces, carbon–hydrogen interactions, π–sigma, π–cation, π–anion, and π–alkyl interactions (Figure 6). α-glucopyranose primarily formed hydrogen bonds with polar residues such as Asp215, Asp352, His351, Glu277, and Arg442, indicating polar-driven binding. Quercetin exhibited the most extensive interactions, involving both hydrogen bonding and π–π stacking with residues including Asp215, Asp352, Arg213, Arg442, Glu277, and Tyr72, suggesting the strongest binding affinity. Parasitic acid showed moderate interactions with Asp215, Asp352, Tyr72, His351, and Glu277, supported by limited hydrophobic contacts. Collectively, quercetin demonstrated the highest binding stability.

## 3. Materials and Methods

The methods used in this study include plant material collection, extraction, isolation, and purification of the novel compound, as well as its structural elucidation using various spectroscopic techniques. The cytotoxic activity of the isolated compound was evaluated against the MCF-7 breast cancer cell line. Additionally, the antioxidant and α-glucosidase inhibitory activities of the compound were assessed. Each of these methods will be described in detail in the subsections below.

### 3.1. Chemicals

Column chromatography was performed using Merck silica gel 60 (Darmstadt, Germany, 230–400 mesh). Thin-layer chromatography (TLC) was carried out on precoated Merck Kieselgel 60 F_254_ plates (0.25 mm), with all solvents (hexane, ethyl acetate, acetone, methanol, chloroform) distilled prior to use in extraction and chromatographic procedures.

### 3.2. Instrumentation

^1^H and ^13^C NMR spectra (including HMQC and HMBC) were recorded on a JEOL JNM-EX500 FT-NMR spectrometer (Akishima, Japan) operating at 500 MHz for ^1^H and 125 MHz for ^13^C-NMR, respectively, using tetramethylsilane (TMS) as an internal standard. Mass spectrometry was performed on a Mariner Biospectrometry instrument (Thermo Fisher Scientific, Waltham, MA, USA) using electrospray ionization (ESI) in positive ion mode. FTIR was carried out using Bruker-Tensor II (Billerica, MA, USA), with a range of 4000–500 cm^−1^ and a resolution of 4.0 cm.

### 3.3. Plant Materials

The leaf of *D. parasiticum* was collected from Bali Botanical Garden plants collection with code XIV.A.12. The plant specimen was identified and deposited at Bali Botanical Garden Herbarium, BRIN Bali, Indonesia.

### 3.4. Extraction

The quantity of 250 g of air-dried leaves of *D. parasiticum* was ground to powder and extracted exhaustively with three different solvents, with hexane, ethyl acetate and methanol, for three extractions each, successively at room temperature. Each extract was collected, filtered and concentrated by rotary evaporation (IKA Rotary Evaporator RV 10, Staufen im Breisgau, Germany) to obtain crude extract. The total extracts obtained from the solvents n-hexane, ethyl acetate and methanol were 5.1 g (2.05%), 37.1 g (14.84%) and 22.4 g (8.96%), respectively.

### 3.5. Isolation and Purification

The ethyl acetate extract (35 g) was subjected to column chromatography and eluted with a hexane-ethyl acetate gradient, yielding 30 primary fractions. These were combined based on their TLC profiles into twelve main fractions: F1 (28.2 mg), F2 (285.7 mg), F3 (174.8 mg), F4 (362.6 mg), F5 (201.9 mg), F6 (574.4 mg), F7 (1778.6 mg), F8 (7637.9 mg), F9 (3812.5 mg), F10 (10,282.6 mg), F11 (3322.8 mg), and F12 (5261.8 mg).

Fraction F12 (5261.8 mg) was subjected to flash chromatography eluting with a gradient of 1:9; 2:8, 3:7, 4:6, 0:10 hexane/ethyl acetate to obtain eight major sub-fractions; F12.1 (141.2 mg), F12.2 (49.5 mg), F12.3 (19.2 mg), F12.4 (269.4 mg), F12.5 (1024.0 mg), F12.6 (1478.3 mg), F12.7 (48.8 mg), and F12.8 (948.5 mg).

F12.4 (269.4 mg) was further purified through Sephadex LH-20 column chromatography using 1:1 chloroform/methanol mixture to yield 43.9 mg of white powder in pure compound. This compound was identified as parasitic acid, and the details of the structural elucidation are included in the Supporting Information.

### 3.6. Cell Culture and Cytotoxic Activity

The cytotoxic activity was assessed using the MCF-7 breast cancer cell line [51]. Cells were maintained in RPMI-1640 medium (GIBCO, Waltham, MA, USA) supplemented with 10% (*v*/*v*) fetal bovine serum (Sigma, St. Louis, MO, USA) and 1% antibiotic-antimycotic (GIBCO) at 37 °C in a 5% CO_2_ atmosphere. For the assay, cells were seeded into 96-well plates at a density of 5 × 10^4^ cells/mL and allowed to adhere for 24 h. After treatment with the test samples, cell viability was determined using the Alamar Blue assay (Bio-Rad Laboratories, Hercules, CA, USA). Following a 3 h incubation with 10 μL of Alamar Blue reagent, fluorescence was measured (excitation 560 nm/emission 590 nm).

### 3.7. DPPH Free Radical Scavenging Activity

The antioxidant activities of *D. parasiticum* leaf extracts were evaluated according to the method of Yen and Chen [52]. Aliquots of samples (2 mL) at different concentrations were combined with 0.5 mL of 1 mM DPPH in methanol. All of the combinations were vigorously shaken and allowed to stand at room temperature for 30 min in the dark. The absorbance of the reaction solutions was measured at 517 nm using a spectrophotometer (UV-VIS Thermo Genesys 30, Waltham, MA, USA). The reference standard used was ascorbic acid and the experiment was performed in triplicate. The IC_50_ value was the effective concentration at which 50% of the DPPH radicals were scavenged and was obtained by interpolation with linear regression analysis. A lower IC_50_ value indicates greater antioxidant activity, with the reducing activity being calculated with the following equation:

(1) $$\%Reduction\;activity=\frac{\left({A_{blank}-A}_{sample}\right)}{\begin{array}{c} A_{blank} \end{array}}\times 100\%$$

where A_blank_ is the absorbance without the sample and A_sample_ is the absorbance of the sample. The IC_50_ value is obtained when the %Reduction activity is 50% based on a calibration curve.

### 3.8. α-Glucosidase Inhibitory Activity

The α-glucosidase inhibitory activity was assessed according to the method of Kim [53] with slight modifications. Briefly, 5 μL of the test sample (dissolved in DMSO at various concentrations) was mixed with 495 μL of 100 mM phosphate buffer (pH 7.0) and 250 μL of 3 mM ρ-nitrophenyl-α-D-glucopyranoside (ρ-NPG) in a reaction tube. The mixture was pre-incubated at 37 °C for 5 min. The enzymatic reaction was then initiated by adding 250 μL of α-glucosidase solution (0.065 U/mL) and incubating at 37 °C for 15 min. The reaction was terminated by adding 1 mL of 0.2 M sodium carbonate (Na_2_CO_3_). The α-glucosidase activity was determined by measuring the release of ρ-nitrophenol at 400 nm. A blank for each sample was prepared by replacing the enzyme solution with phosphate buffer to correct for non-enzymatic hydrolysis. The inhibition percentage was calculated using the following formula:

(2) $$\%Inhibition=\frac{\left(A_{blank}-A_{sample}\right)}{\begin{array}{c} A_{blank} \end{array}}\times 100\%$$

where A_blank_ is the absorbance without the sample and A_sample_ is the absorbance of the sample. The IC_50_ is estimated based on a calibration curve where the %Inhibition is 50%. The experiments were performed in triplicate, and the data are shown as mean values ± SDs. Quercetin was used as a standard.

### 3.9. Molecular Docking

The crystal structures of the complex Epidermal Growth Factor Receptor (EGFR) (PDB ID: 4HJO) were obtained from the RSCB Protein Data Bank (www.rcsb.org). Chimera 1.1.3 software was used to create protein and natural ligand structures, which were saved as PDB files [54]. Compound structures were generated using Avogadro 1.2.0 software, optimized with Orca 4.2 software using the DFT B3LYP 6-31G technique, and stored in.pdb format [55]. AutoDockTools 1.5.6 was used for docking simulations and redocking of the EGFR protein in a 40 × 40 × 40 Å grid box in x, y, and z dimensions [56]. The Lamarckian Genetic Algorithm (LGA) approach was used for the 50 runs, and the molecular docking results were then shown using Discovery Studio Visualizer 17.2.0 2019 [57].

## 4. Conclusions

This study reports on the successful isolation and structural elucidation of parasitic acid, a novel trinorditerpene, from *Dysoxylum parasiticum* leaves. This compound demonstrated remarkable multi-target therapeutic potential through three distinct biological activities: cytotoxicity against MCF-7 breast cancer cells (IC_50_ 29.0 ± 0.8 μg/mL), DPPH radical scavenging activity (IC_50_ 10.91 ± 0.04 μg/mL), and α-glucosidase inhibition (IC_50_ 36 ± 1 μg/mL). The unique structural features of the compound, including a trinorditerpenes skeleton with conjugated double bonds, an electron-rich furan ring, and carboxylic acid groups, likely contribute to its diverse biological activities through multiple mechanisms of action.

## Figures and Tables

**Table 1:** Cytotoxic activity assay. Different letters indicate significant difference (*p* < 0.05).

Compound	Concentration (µg/mL)	% Inhibition				IC_50_ (µg/mL)
		1	2	3	Average	
Erlotinib	40	99.73	98.83	98.64	99.1 ± 0.5	6.6 ± 0.4
	20	87.21	86.26	87.56	87.0 ± 0.6	
	10	63.19	61.02	62.12	63.0 ± 0.9	
	5	33.74	32.83	33.63	33.0 ± 0.4	
Parasitic acid	100	92.6	93.1	92.8	92.8 ± 0.3	29.0 ± 0.8
	50	70.8	71.0	70.2	70.7 ± 0.4	
	25	52.0	50.3	51.9	51.4 ± 1.0	
	12.5	32.7	31.2	31.5	31.8 ± 0.8	

**Table 2:** The results of the molecular docking analysis of compound parasitic acid against EGFR proteins.

Compound	Binding Energy (kcal mol^−1^)	Hydrogen Bond
Erlotinib	−8.27	Lys705, Met769, Cys773
Parasitic acid	−8.18	Met769

**Table 3:** The results of DPPH free radicals scavenging activity.

Compound	Concentration (µg/mL)	%Reduction Activity	IC_50_ (µg/mL)
Quercetin	1	6.91	6.1 ± 0.1
	2.5	17.30	
	5	56.82	
	10	94.66	
Parasitic acid	5	26.58	10.91 ± 0.04
	10	53.51	
	25	92.93	

**Table 4:** α-Glucosidase enzyme activity inhibition test results.

Compound	Concentration (µg/mL)	%Inhibition	IC_50_ (µg/mL)
Quercetin	1	46.84	1.29 ± 0.06
	2.5	58.89	
	5	73.87	
	10	86.16	
Parasitic acid	5	0.39	36 ± 1
	10	2.32	
	20	15.12	
	25	28.18	

**Table 5:** The results of the molecular docking analysis of parasitic acid against *S.cerevisiae* isomaltase proteins.

Compound	Binding Energy (k cal mol^−1^)	Hydrogen Bond
α-glucopyranose	−6.99	Asp69, His112, Asp215, Arg213, Glu277, His351, Asp352, Agr442
Quercetin	−7.16	Arg213, Asp215, Glu277, Gln279, Glu411
Parasitic acid	−5.28	Tyr72, Asp215

## Data Availability

Dataset available on request from the authors.

## Supplementary Materials

The following supporting information can be downloaded at: https://www.mdpi.com/article/10.3390/molecules30244747/s1, Details of the extraction, fractionation and purification are provided. In addition, structure elucidation based on various experimental spectra and their interpretation is included.
