# Supplementary material for: Novel Trinorditerpene from Dysoxylum parasiticum (Osbeck) Kosterm: Leaf Extract with Cytotoxic, Antioxidant and α-Glucosidase Inhibitory Activities
Source: Molecules. 2025 Dec 12;30(24):4747. doi: 10.3390/molecules30244747 (PMC12735834; doi:10.3390/molecules30244747)
Supplement: Supplementary file 1 [file molecules-30-04747-s001.zip › molecules-3962522-supplementary.pdf]

Supporting Information

Extraction, Fractionation and Purification

Sequential extraction of dried *D. parasiticum* leaves (250 g) with hexane, ethyl acetate, and methanol yielded crude extracts of 5.1 g (2.05%), 37.1 g (14.84%) and 22.4 g (8.96%), respectively. The ethyl acetate extract showed promising activity in preliminary bioassays and was selected for further fractionation. Column chromatography of the ethyl acetate extract (35 g) using a hexane-ethyl acetate gradient yielded twelve combined fractions (F1-F12). Fraction F12 (5.26 g) demonstrated the highest bioactivity and was further separated by flash chromatography using hexane/ethyl acetate gradients (1:9 to 0:10) to obtain eight major sub-fractions (F12.1-F12.8). Sub-fraction F12.4 (269.4 mg) was purified through Sephadex LH-20 column chromatography using chloroform/methanol (1:1) to yield parasitic acid (43.9 mg) as an amorphous white powder. The structure was established through comprehensive spectroscopic analysis, including 1D and 2D NMR spectroscopy and high-resolution mass spectrometry as described below.

Fourier-Transform Infrared Spectroscopy/FTIR

The peak at 1666.21 cm^{-1} is in the range 1610-1680 cm^{-1}, corresponding to the alkene (C=C). The peak at 2918.05 cm^{-1} is in the range 2850-2970 cm^{-1} and is associated with C-H bonds. The peak at 3252.28 cm^{-1} is in the range 3200-3600 cm^{-1} and assigned as the O-H functional group.

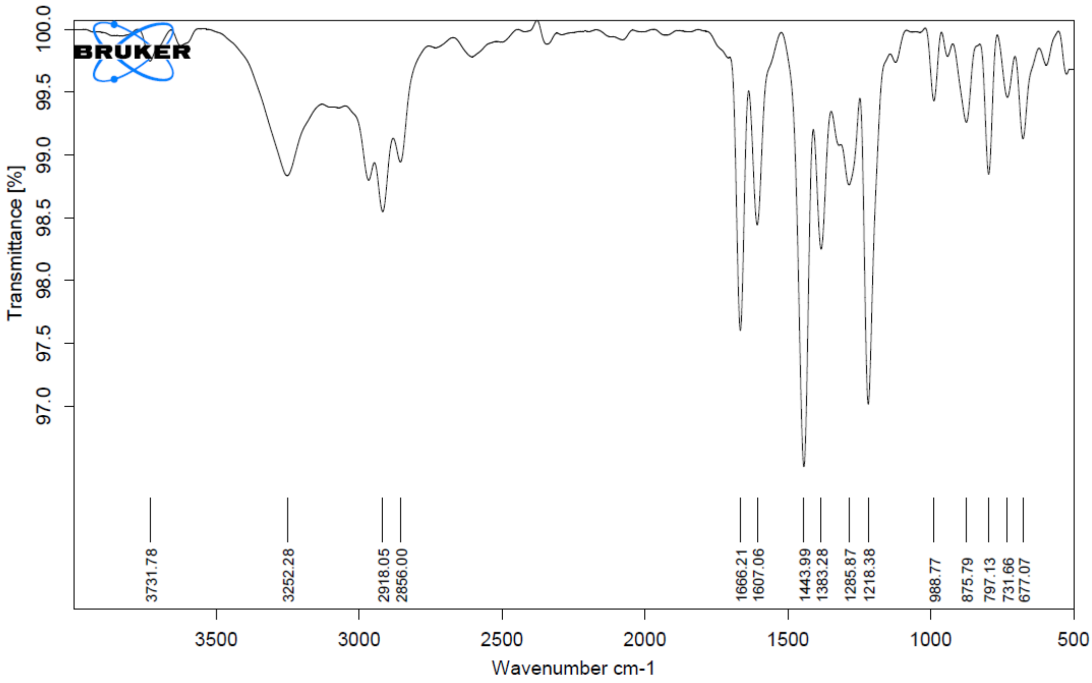

Figure S1. FTIR results of the compound

Structure Elucidation

The compound was isolated as an amorphous white powder, and based on the ^{1}H, ^{13}C, DEPT, and HMQC spectra, the following functional groups were detected 3 × CH_3, 3 × CH_2, 4 × =C-H, 6 × -C= and 1 × -COOH. The compound was predicted to have a chemical formula: $C_{17}H_{20}O_3$ and an exact mass of 272.141245 Da. This result was also verified by HR-LC-MS/MS measurement, the results yielded the same chemical formula $C_{17}H_{20}O_3$ and an exact mass of 272.14124 Da.

The ^{1}H-NMR, ^{13}C-NMR, DEPT-135, HMQC, and HMBC were measured and the respective spectra are included in Figures S2 to S4.

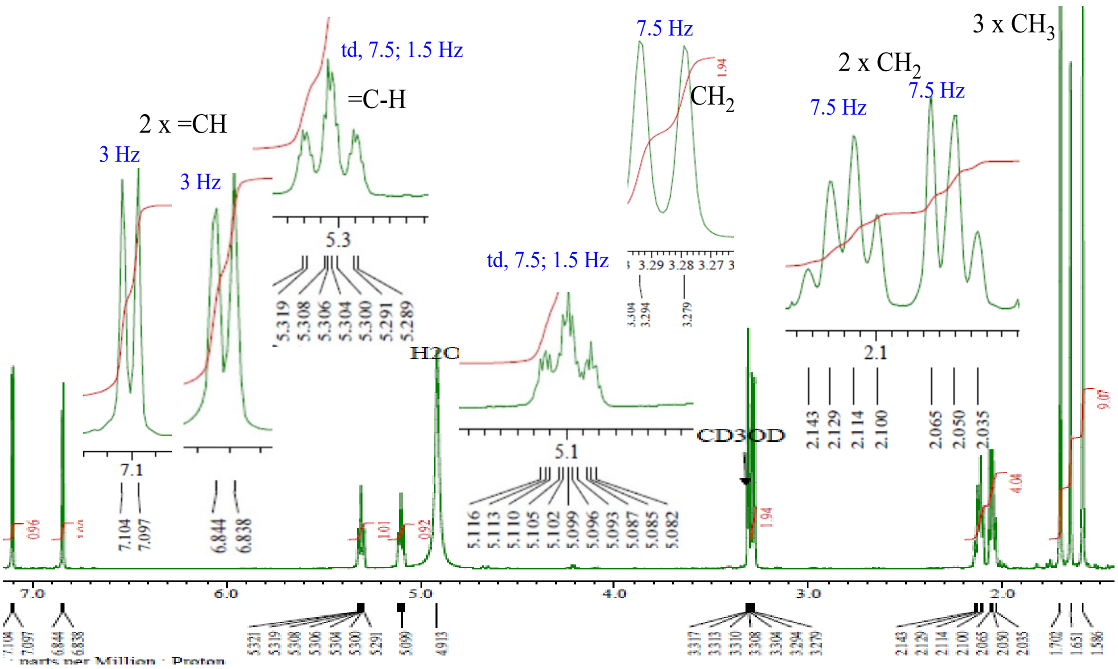

Figure S2. ^{1}H-NMR spectrum of the compound (500 MHz), in CD_3OD solvent.

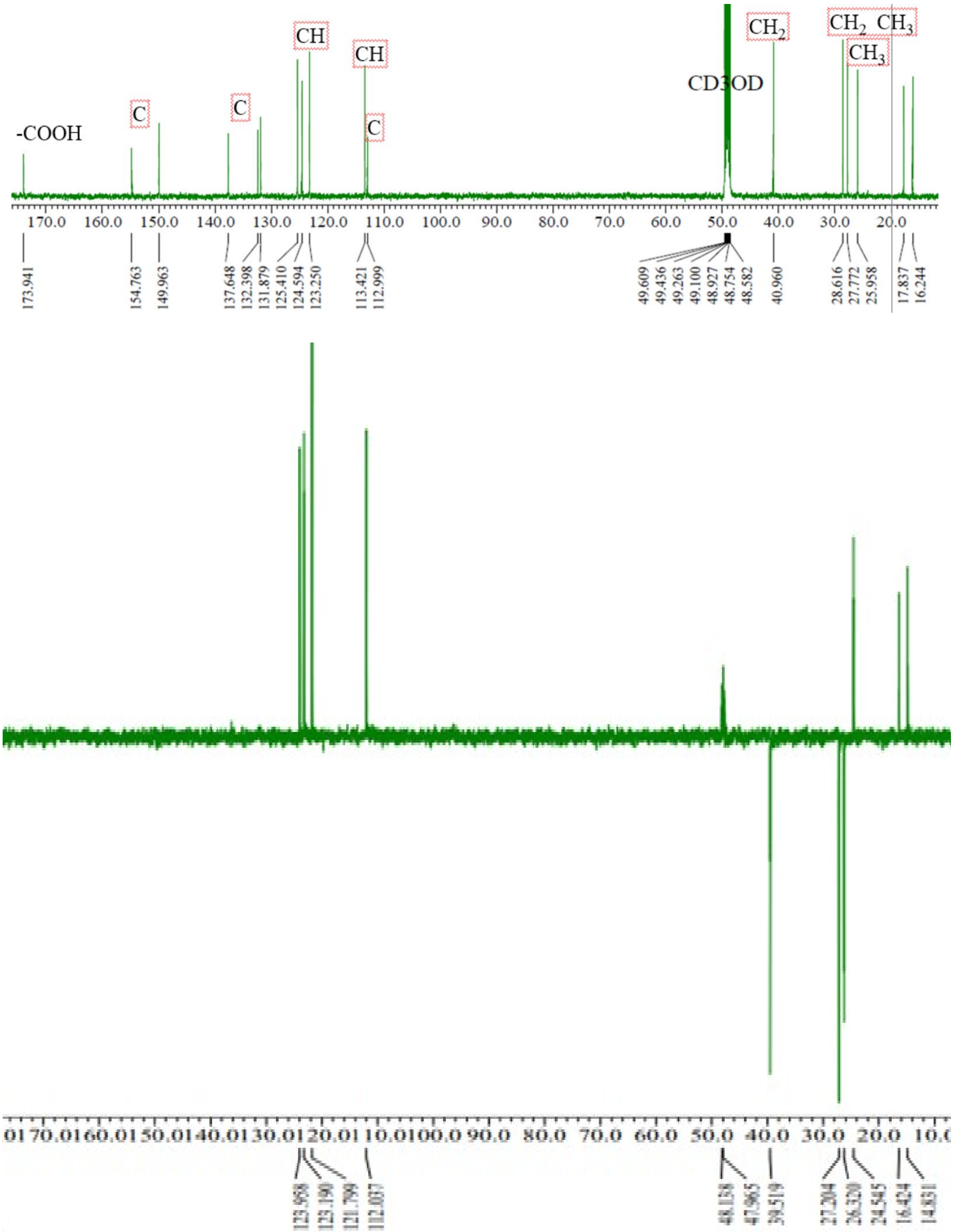

Figure S3. ^{13}C-NMR and DEPT-135 spectrum (125 MHz, in CD_3OD).

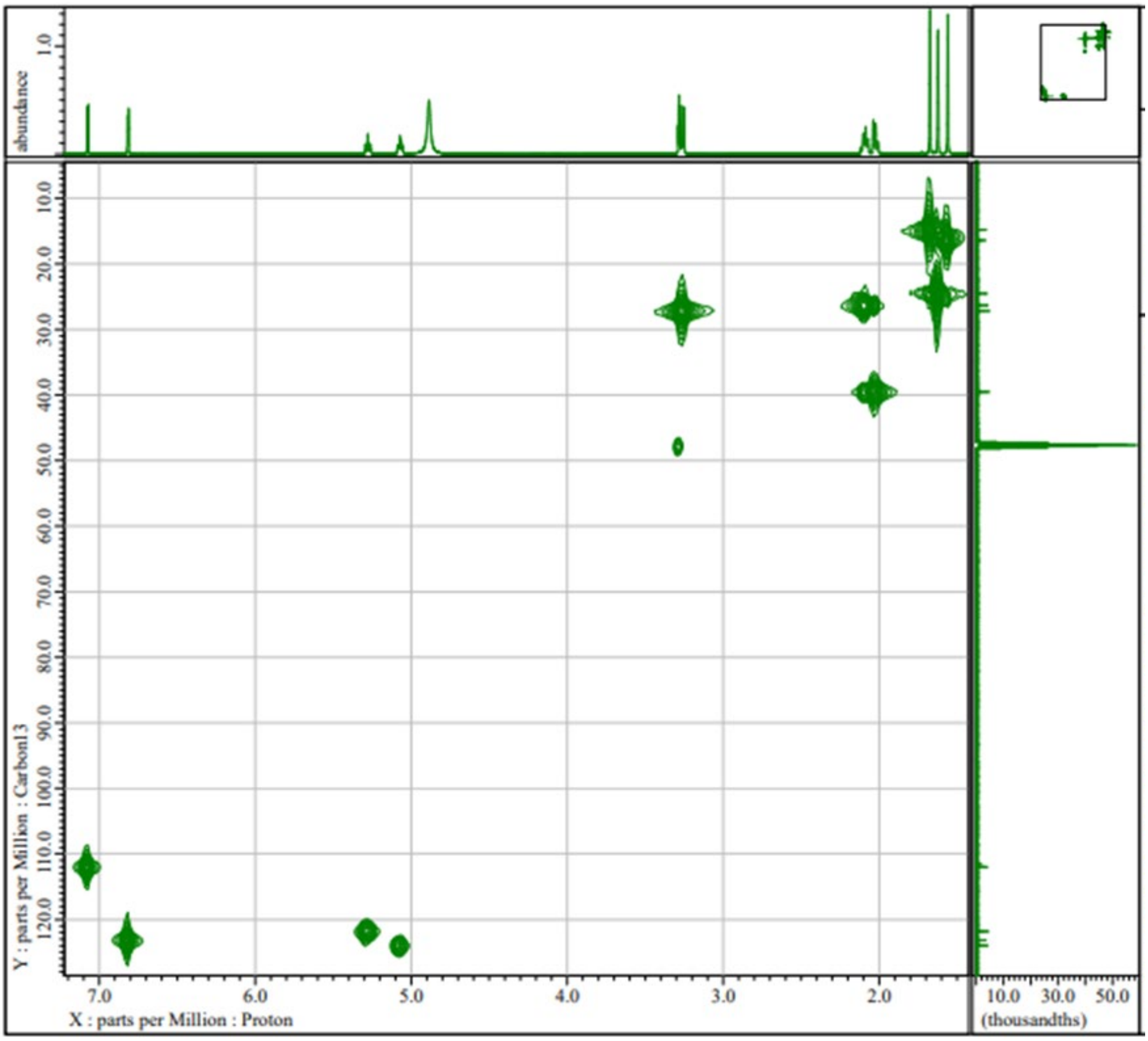

Figure S4. H and C correlation spectra (HMQC).

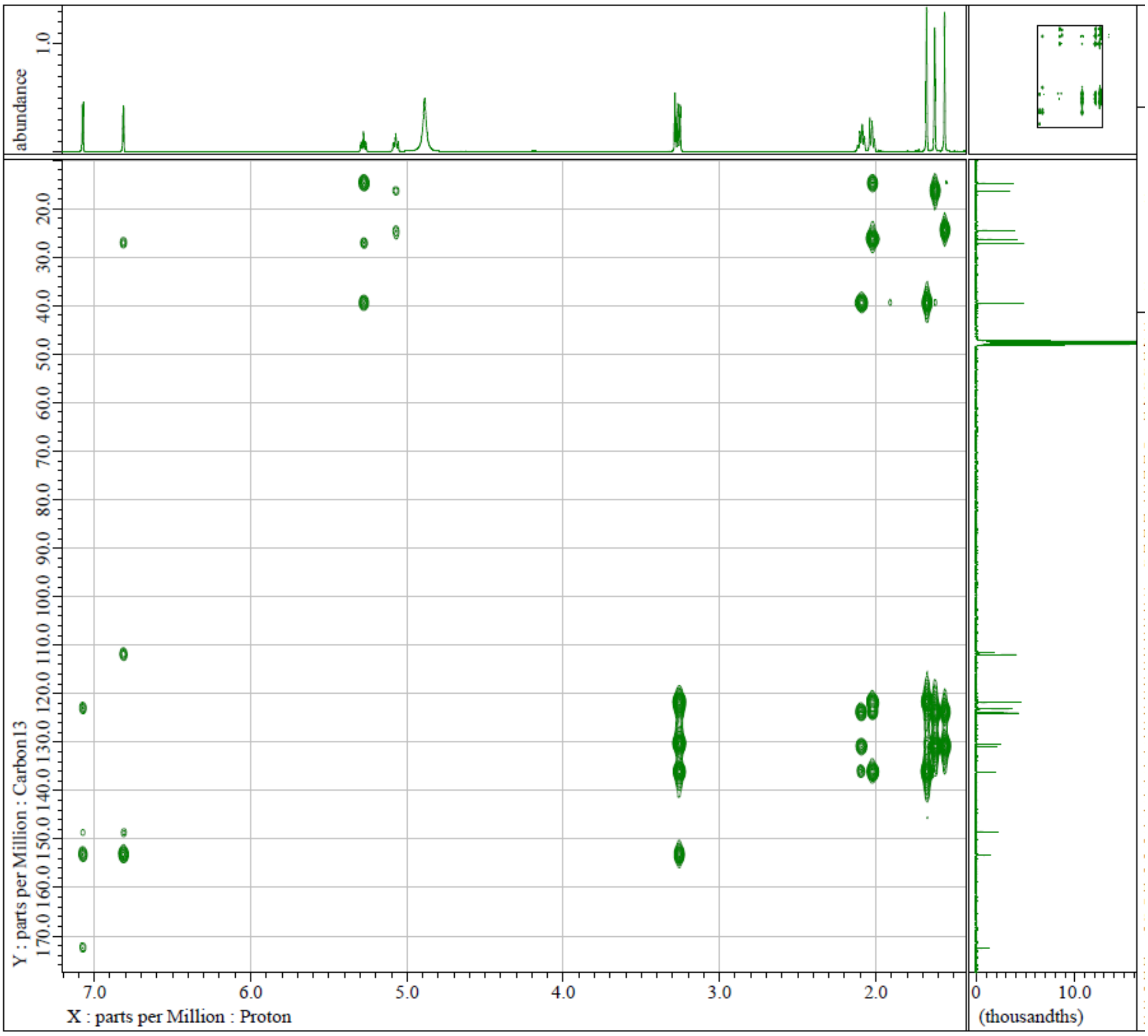

Figure S5. H and C correlation Spectra (HMBC)

The ^{1}H-NMR spectrum of this compound shows the presence of a specific signal from 2 protons in the form of a doublet (d) with a value of J is 3 Hz at d_H 6.84 ppm (d, H-11) and 7.1 ppm (d, H-12), which is a furan ring, and binds to a CH_2 group at d_H 3.29 ppm (2H, H-9, d, 7.5 Hz). The signals at δ 5.1 and 5.3 ppm appeared as broad triplets (br t, J ≈ 7.5 Hz), consistent with vinylic or olefinic protons exhibiting partial coupling overlap. Moreover, there is a signal from 3 methyl groups (s), seen at relative downfield area d_H 1.59 ppm (3H, s, H-17); 1.65 ppm (3H, s, H-16) and 1.70 ppm (3H, s, H-15), indicating that this methyl group is attached to a double bond. The other signals at d_H 2.05 ppm (2H, d, 7.5 Hz, H-7); 2.13 ppm (2H, d, 7.5 Hz, H-4) were identified as methylene (CH_2). These results are strengthened by the ^{13}C-NMR spectrum, as shown in the structural image of Figure S6, where the signal at 173.94 ppm (s, C-14) indicated the presence of a carboxylate functional group (-COOH). The chemical shift of furan ring was 154.76 ppm (s, C-10); 149.96 ppm (s, C-13); 124.59 ppm (d, C-12) and 113.42 ppm (d, C-12), which are shown in Figure S3 for more details.

Based on the measurement results of the H and C-NMR spectra and supported by the HMQC spectrum, the measured chemical shift values are shown in Figure S6 and summarized in Table S1. To ascertain the position of the chemical shift value and the position of each group, the long-range correlation spectrum (1-4 bonds) of HMBC was measured, as shown in Figure S5. The position of the carboxylic group (-COOH) is placed at C-13 of the furan ring because of the presence of long-range coupling, correlating between the signal d_H 7.1 ppm (d, 3 Hz) and d_C 173.74 ppm. The position of the methyl signal at d_H 1.70 ppm is placed at C-9, supported by HMBC correlation data with d_C 137.05 ppm (C-8) and 40.96 ppm (C-1), as summarized in the structure below. Based on the above chemical shift data summarized in Table S1 below, this newly identified compound, named in this study as parasitic acid, is a novel trinorditerpene with the systematic name 12-(3-carboxyfuran-2-yl)-trinorlabda-5, 9-dien-15-oic acid.

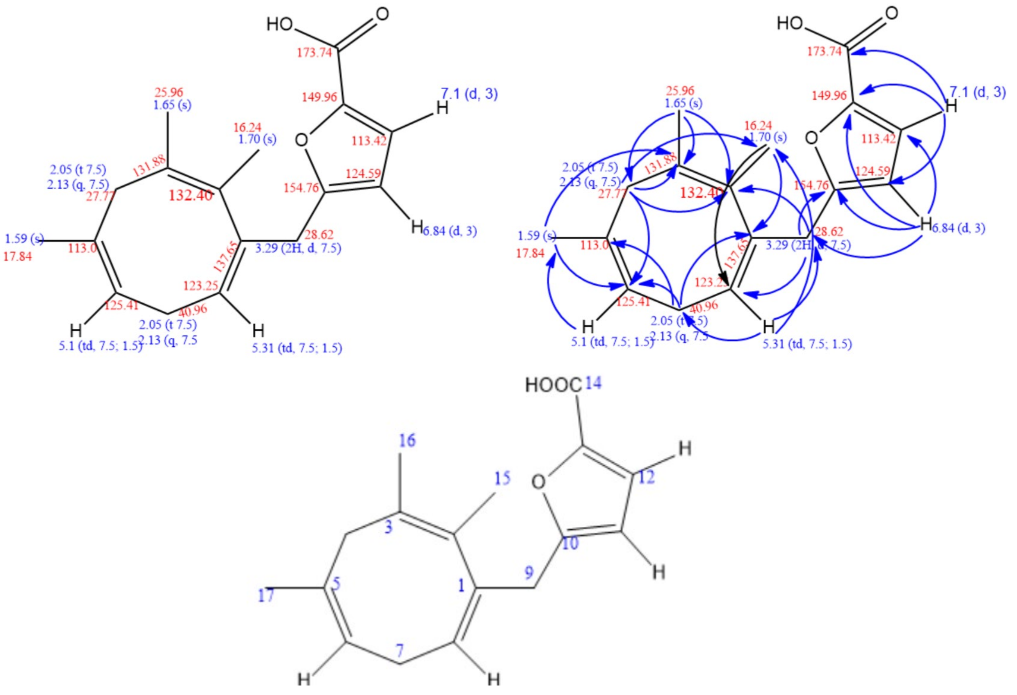

Figure S6. The chemical structure of the **parasitic acid**, the chemical shift, and the numbering of its carbon atoms.

Table S1. Chemical shifts of H (500 MHz) and C-NMR (125 MHz) data for **parasitic acid** (CD_3OD, JEOL JNM 500).

C No	H-NMR (d in ppm, m, J in Hz)	C-NMR (d)*	
1		137.65	C
2		132.40	C
3		131.88	C
4	2.05 (t, 7.5)<br>2.13 (q, 7.5)	27.77	CH_2
5		113.00	C
6	5.1 (t, 7.5; 1.5)	125.41	CH
7	2.05 (t, 7.5)<br>2.13 (q, 7.5)	40.96	CH_2
8	5.31 (t, 7.5; 1.5)	123.25	CH
9	3.29 (2H, d, 7.5)	28.62	CH_2
10		154.76	C
11	6.84 (d, 3)	124.59	CH
12	7.1 (d, 3)	113.42	CH
13		149.96	C
14		173.74	COOH
15	1.70 (s)	16.24	CH_3
16	1.65 (s)	25.96	CH_3
17	1.59 (s)	17.84	CH_3

*H and C correlation determined based on HMQC spectra
